# Prototype master protocol for benchmarking of real‐world follow‐up data in glaucoma

**DOI:** 10.1111/aos.17453

**Published:** 2025-02-13

**Authors:** Pekko Hujanen, Anja Tuulonen, Sanna Leinonen, Kristian Vepsäläinen, Gauti Jóhannesson, Anu Vaajanen, Eemil Lehtonen, Hannele Uusitalo‐Jarvinen

**Affiliations:** ^1^ Tays Eye Centre Tampere University Hospital Tampere Finland; ^2^ Faculty of Medicine and Health Technology Tampere University Tampere Finland; ^3^ Department of Clinical Sciences, Ophthalmology Umeå University Umeå Sweden; ^4^ Wallenberg Centre for Molecular Medicine Umeå University Umeå Sweden; ^5^ Department of Ophthalmology University of Iceland Reykjavik Iceland; ^6^ Eye and Vision Research SILK, Faculty of Medicine and Health Technology Tampere University Tampere Finland

**Keywords:** benchmarking, glaucoma, master protocol, progression, real‐world data

## Abstract

**Purpose:**

The purpose of this study was to create a prototype master protocol for benchmarking glaucoma real‐world data (RWD). Benchmarking is part of the digital innovation strategy of the Finnish *aces‐rwm* ecosystem (automation of care and evaluation of the system with real‐world monitoring).

**Methods:**

We collected glaucoma RWD in 2012–17 at Tampere University Hospital (Tays) and compared them to six published RWD sets (one in Sweden and five in England). Visual field (VF) data at Tays were retrieved from the perimeter, and clinical RWD were collected manually. At baseline, VF data were available in 2511 of 4121 glaucoma patients (61%), of whom 1413 patients (56%) had 5 years of VF follow‐up by 2017 (34% of all 4121 patients). Mean deviation (MD) data were analysed in multiple ways, considering also age and intraocular pressure (IOP).

**Results:**

In most data sets, higher age was related to faster progression. At Tays, the distributions of progression rates were similar in better and worse eyes. The proportions of eyes at Tays with the medium rate of MD progression (17% for 0.5–1.5 dB/year) and the fast rate (6% for >1.5 dB/year) were similar to the published RWD trial in England. The published datasets show significant variability in how their findings were reported.

**Conclusions:**

This study represents a first step toward the development of a master protocol for real‐world benchmarking of glaucoma care. Further refinement of the protocol will encourage and require national and international collaboration in order to produce comprehensive and comparable real‐world EHR data sets.

## 1 INTRODUCTION

All five Finnish university eye clinics have proactively and systematically collaborated for over 20 years in developing national policies and approaches to provide equal and the best possible eyesight and well‐being with the available resources in eye care (Table 1). Despite the best of intentions, the productivity benchmarks between the university eye clinics in 2012–2017 continued to indicate large variability in care processes and costs without understanding their impact on patient outcomes. Consequently, the Finnish university eye clinics decided to create an ecosystem for measuring real‐world cost‐effectiveness of the ‘Big Four’ eye diseases, which affect two‐thirds of their patients, visits, and costs, i.e., age‐related macular degeneration (AMD), glaucoma, diabetic retinopathy, and cataract (Tuulonen et al., 2022). The ecosystem is called *aces‐rwm* (*automation in care and evaluation of system with real‐world monitoring*) and consists of three components: (1) principles to deal with increasing demand and limited resources, (2) routine collection of structured Real‐World Data (RWD) using the ‘Big Four’—specific structured electronic health records (EHR), including also measures of health‐related quality of life (QoL) and costs, and (3) evaluation and benchmarking real‐world outcomes and cost‐effectiveness (Tuulonen et al., 2022).

**TABLE 1:** Background, consensus processes, and involved parties leading to the stage of the current study since 2002.

2002–24	All four editions of the publicly funded Finnish Current Care Glaucoma Guidelines (Leinonen et al., 2024; Tuulonen et al., 2003) have been developed under the leadership of the Finnish Medical Society Duodecim in collaboration with the Finnish Ophthalmological Society and the Finnish Glaucoma Society
2004	Finnish Current Care Guidelines form the basis for national access to care criteria, first decreed in 2004. The eye care criteria, including glaucoma (Tuulonen et al., 2009), are defined by all chief physicians of public eye clinics and decreed by the Ministry of Social Affairs and Health with the latest update in 2019
2009–11	New eye care strategy developed for Tays Eye Centre: (1) Identification of the high‐volume ‘Big Four’ eye diseases (age‐related macular degeneration, glaucoma, diabetic retinopathy, and cataract) comprising 2/3 of eye care (patients, visits, and costs); (2) Stratification and prioritisation of patients based on permanent visual loss; (3) standardisation of services for low‐risk patients; (4) maximising productivity; and (5) Implementing shared care (Tuulonen et al., 2016)
2012	New building opened for Tays Eye Centre. In 2011–15, the implementation of the new strategy produced a 46% increase of services with a 15% increase in the work force (Tuulonen et al., 2016). The nurses were trained to enter the clinical data manually into a structured paper data collection form, to pre‐evaluate the digital photographs of the retinal nerve fibre layer and optic nerve head, as well as visual field tests to be checked by a glaucoma specialist
2012–17	Two productivity benchmarks between university eye clinics revealed huge variabilities in care practices and costs without understanding their impact on outcomes (data on file), with the shared agreement that all cannot be right (Tuulonen et al., 2022). Structured glaucoma RWD was collected manually in Tays Eye Centre
2016–21	Tays Eye Centre was awarded 1‐star Reference Site status for the P5SE model in 2016–18 and 3‐star Reference Site status for the *aces‐rwm* ecosystem in 2019–21 by EI PAHA (European Innovation Partnership on Active and Health Ageing) of the European Commission within the B3 Action Group (Bousquet et al., 2019)
2016–18	Development of a structured glaucoma‐specific EHR prototype RWD collection tool in Tays exploiting the experiences of the manual structured data collection in 2012–17
2019	The first full‐year RWD using a structured EHR prototype for glaucoma (Sulonen et al., 2024) with data reported using the CODE‐EHR best‐practice framework (Kotecha et al., 2022)
2019–23	In collaboration with all university eye clinics, the 2009 Tays strategy was expanded to a published *aces‐rwm* ecosystem for measuring real‐world cost‐effectiveness in eye care (Tuulonen et al., 2022). The joint procurement signed by all university hospitals tendered the development of structured EHR‐RWD collection and evaluation tools for the ‘Big Four’ eye diseases, supported by the government organisation Business Finland
	The *aces‐rwm* EHR tool package includes the development of benchmarking tools to be produced automatically by the EHR. The 2019 Finnish Act on the Secondary Use of Social and Health Data allows benchmarking between units only using aggregated data, i.e., groups of individuals in such a way that the individuals cannot be identified
	Since 2023, the largest‐ever national health care reform in Finland emphasises cost‐effectiveness and knowledge management. Wellbeing Services County of Pirkanmaa, including Tays (Tampere University Hospital), is the coordinator of the national effectiveness centre for information, dissemination of data collection, and reporting
2024	In Tays, the prototype EHR‐RWD collection tool for glaucoma (used in 2019–23) was replaced with a new *aces‐rwm*‐compatible, glaucoma‐specific EHR, developed in collaboration with all university eye clinics and ready to be put to use in all of them. The EHR tool package is translatable to any language (currently available in Finnish, Swedish, and English)
Current stage	To promote and inspire international collaboration and gain feedback for the development of the reporting benchmark protocol, the current study: (1) reports Tays RWD in 2012–17, (2) compares these data to the published RWD data sets from two publicly funded health care systems, (3) based on steps 1 and 2, presents a prototype master protocol for RWD benchmarking to be developed further, and (4) publishes it (as preferred by CODE‐EHR for structured electronic health‐care records, Kotecha et al., 2022) (Data S1)
Next stage	For further development of the benchmarking protocol: (1) the cross‐sectional Tays 2019 EHR‐RWD (Sulonen et al., 2024) will be compared to the 2019 RWD collected in Umeå, Sweden, and (2) Tays 2012–17 of this study will be compared to the structurally recorded EHR‐RWD collected in 2019–23

In 2019, all Finnish university hospitals signed a joint procurement document that called for bids on the development of structured, bespoke, and *aces‐rwm* ‐compatible EHR‐RWD collection and evaluation tools for the ‘Big Four’ eye diseases (Tuulonen et al., 2022). The development in 2019–23 was supported by Business Finland, an organisation within the Finnish government. During the development of the structured EHR‐RWD collection tool for glaucoma, the manual data collected in 2012–17 (current study) and the EHR prototype used at Tays Eye Centre (Tays) in 2019–23 (Sulonen et al., 2024) were utilised in the development of the new, consensus‐based bespoke EHR‐RWD collection tools, first put to use for glaucoma at Tays in 2024 (Table 1).

The requirement for collecting standardised data to aid decision‐making is not new to medicine. For example, in 1987 Yates concluded that health care authorities need to know what work is being done, what information‐gathering methods should be improved, and what definitions should be used (Yates, 1987). In addition, the Joint Commission Resources Inc. (2012) reported that organisations complying with national requirements must be able to benchmark their patient outcomes to other organisations. The best‐practice framework for the use of structured EHR in clinical research (CODE‐EHR) (Kotecha et al., 2022) recommends published protocols for contributing to the credibility and reproducibility of the EHR‐based studies (Table S1), also in glaucoma (Higgins et al., 2024).

Prior to the availability of EHR‐RWD from other Finnish university eye clinics, the purpose of this study was to compare the glaucoma RWD collected at Tays to published data sets in Sweden and England to aid the development of a prototype master protocol for *aces‐rwm* benchmarking. The current study also serves to inform potential international collaborators of the availability of Finnish glaucoma RWD for benchmarking and to invite international collaboration for further development of benchmarking protocols.

## 2 MATERIALS AND METHODS

The *aces‐rwm* ecosystem's structured benchmarking platform for the ‘Big Four’ eye diseases is a key factor in its digital innovation strategy to evaluate the real‐world outcomes and cost‐effectiveness of eye care (Table 1; Tuulonen et al., 2022). To the best of our knowledge, the concept of a master protocol defining how to use RWD from routine care for benchmarking purposes has not been reported for any of the ‘Big Four’ eye diseases.

The US National Institutes of Health (2024) defines a master protocol as a trial design that can test multiple subpopulations in parallel , without a need to develop new protocols for every study. Similarly, Franklin et al. (2024) describe their vision for modernised data infrastructure: (1) clinical trial design using complex elements, such as master protocols and adaptive platform design (in *aces‐rwm*, the RWD benchmarking platform for the ‘Big Four’), (2) reusing data for multiple purposes, e.g., for addressing more than one research question or study, (3) using these kinds of RWD to inform different types of decisions made by different evaluators of the evidence (in *aces‐rwm*, the cost‐effectiveness of eye care), and (4) using streamlined RWD collection from routine care (in *aces‐rwm*, the structured ‘Big Four’ bespoke electronic health records).

This study focuses on developing a prototype master protocol for the *aces‐rwm* benchmarking platform by using glaucoma RWD collected at Tays in 2012–17 and comparing them to published studies. Tays Eye Centre, Tampere University Hospital, Finland, is the only publicly funded eye clinic providing services for the population of 0.56 million in Pirkanmaa Wellbeing Services County, which represents 10% of the 2024 population of Finland. Tays organises glaucoma care for almost 90% of patients using reimbursed glaucoma medications in its serving area (Sulonen et al., 2024).

The a*ces‐rwm* strategy identifies glaucoma as one of the four high‐volume eye diseases requiring stratification and prioritisation of services to prevent visual disability (Table 1) (Tuulonen et al., 2022). The opening of the new building for eye care at Tays in 2012, including its new care delivery strategy, represented a kick‐off for rapid development of care processes (Kokkinen & Lehto, 2011; Tuulonen et al., 2016). To start with, a structured paper‐based data collection form for glaucoma was developed into which nurses were trained to enter the clinical data manually. These data were then entered into the hospital's unstructured electronic health record. In 2016–18, manual structured data collection was developed further via the implementation of a bespoke, glaucoma‐specific, structured prototype EHR, which was used at Tays from 2019 to 2023. The tendered *aces‐rwm* ‐compatible EHR was developed in collaboration with all Finnish university eye clinics and was first put to use in the Tays glaucoma service in 2024 (Table 1).

The reporting of this article adheres to the CODE‐EHR checklist, which recommends published protocols for (1) dataset construction and data fitting the purpose, (2) definition and analysis of outcome, and 3) ethics and governance (Kotecha et al., 2022; Table S1 with 5 out of 5 preferred standards met in the CODE‐EHR checklist).

### 2.1 Tays dataset construction and data fitting the purpose

Of the 4121 glaucoma patients visiting Tays in 2012, visual fields (VF) were tested in 2511 patients (61%). The rest of the visits in 2012 included measurement of intraocular pressure (IOP) without a VF test. Of the 2511 patients with VFs in 2012, 1413 patients had 5 years of VF data (56%) in 2017 after excluding 92 patients (90 with false positive VF responses ≥15% and two patients younger than 18 years). Overall, of all 4121 patients seen in 2012, 1413 (34%) had 5‐year VF data to be analysed in this study.

We also estimated the reasons for non‐attendance. In 2012, 217 of 4121 (5%) patients examined did not receive a glaucoma diagnosis and had no follow‐up. Approximately 2% of patients each year, i.e., 444 of 4121 (11%) patients between 2012 and 2017, were no‐shows for unknown reasons, or their general condition did not allow VF testing, or the patient had moved from the Tays serving area. According to the publicly available Info‐Tray provided by the Social Insurance Institution of Finland (2024), approximately 4% of glaucoma medication users per year deceased in Pirkanmaa Wellbeing Service County in 2012–17. Thus, we estimated that 983 of the 4121 patients (24%) had died in 2012–17. Overall, of the 2477 patients (=4121‐217‐444‐983) who were alive and available for testing in 2017, 1413 (57%) visited Tays in 2017. Due to Tays' 2‐year follow‐up interval, 1064 (2477–1413) of 4121 (26%) patients did not visit Tays in 2017 and were estimated to be scheduled for a visit in 2018.

The overall costs per glaucoma patient per year, including both out‐ and in‐patient care as well as all glaucoma‐related procedures, were retrieved from the university hospital's financial administrative database, representing total direct costs for glaucoma management from the payer's perspective. The overall yearly costs were divided by the total number of glaucoma patients visiting Tays each year.

Changes in the trends and types of medications, including their costs, will be reported separately using the data from the Social Insurance Institution, Finland, which maintains a registry of use and cost data of all glaucoma medications purchased by patients from pharmacies in Finland and in the Tays area.

### 2.2 Disease definitions

The first Finnish Current Care Guideline for glaucoma was published in 2002 (English translation by Tuulonen et al., 2003) with the latest update in 2023 (English translation by Leinonen et al., 2024). Over the years, the main diagnostic principles have remained the same; that is, glaucoma diagnosis is based on using the ‘2 out of 3’ rule in which at least two concomitant findings in the RNFL (retinal nerve fibre layer), ONH (optic nerve head), and/or VF indicate congruent glaucomatous damage. The guideline recommends taking the RNFL/ONH/VF test set at diagnosis and thereafter every 1–2 years, depending on the patient's risk profile. For example, in 2019 at Tays (Sulonen et al., 2024), 44% of patients were invited to a control visit within 2 years. Event‐based progression analysis rests on clinical evaluation of RNFL, ONH, and VF, i.e., progression in any of the three tests (e.g., a widening of an RNFL defect) flags the need for intensified therapy, considering risk factors, such as patient age, disease stage, and IOP (Leinonen et al., 2024). At Tays, the VF of the right eye has always been examined first.

Tays complies with the recommendations of the Finnish glaucoma guideline and creates for every patient an individual, structured 2‐year surveillance and treatment plan (Leinonen et al., 2024). The plan is delivered to the patient, and in stable glaucoma includes: (1) definition of an optimum target IOP (at least a 25% reduction from untreated IOP), (2) an IOP level that is considered to require intensifying therapy with a plan for the next two treatment options, and (3) specification for the required frequency of IOP measurements. From 2012 onwards, patients have been advised to have their IOPs measured in optician shops using rebound tonometry (Kontiola et al., 2001), which is also used at Tays. If a patient's IOP reaches a level requiring a change in therapy, the patient calls Tays to get their predetermined next intervention, for example, a prescription for a new drug, an appointment for laser therapy, or surgical consultation. For high‐risk glaucoma patients, the Finnish guideline recommends at least a 35% reduction in IOP and proceeding to surgical treatment with a low threshold.

Visual acuity (VA) at Tays is predominantly measured using a Nidek autorefractometer (Nidek Co., LTD, Gamagori, Aichi, Japan), or using a Snellen chart and refraction, habitual correction, or pinhole. Beginning in 2024, Snellen VA is automatically converted by the *aces‐rwm* compatible RWD collection tool (Avenue Flow, Optomed Plc, Helsinki, Finland) into Early Treatment Diabetic Retinopathy Study (ETDRS) letters using the formula 85 + 50 × log (Snellen fraction) and treated as a continuous variable (Gregori et al., 2010). Finger counting vision is recorded as three ETDRS letters, hand movement as two letters, and light perception as one ETDRS letter.

Humphrey VFs were taken using the Swedish Interactive Threshold Algorithm (SITA) Fast 24–2 program (Carl Zeiss Meditec Inc.). Structural damage at Tays is evaluated using ONH and RNFL digital photographs and labelled as glaucomatous, normal, not assessable, or no picture. The ‘2 out of 3’‐analysis (RNFL, ONH, VF) has been structurally recorded at Tays from 2019 onwards after deployment of the prototype glaucoma‐specific structured EHR (Sulonen et al., 2024). In the EHR, progression is categorised as ‘Yes/No/Cannot be assessed/No prior tests’. Optical Coherence Tomography is not used in glaucoma diagnostics and follow‐up at Tays.

### 2.3 Comparison data sets from Sweden and England

At the time of this investigation, glaucoma RWD are not yet available from other Finnish university eye clinics. Therefore, we chose a Swedish study (Heijl et al., 2013) and five English RWD sets for comparison with our findings (Boodhna et al., 2015; Boodhna & Crabb, 2015; Kelly et al., 2019; Kelly et al., 2020; Kirwan et al., 2014). Like Finland, both Sweden and England have publicly funded health care systems and national glaucoma guidelines.

The English datasets partly reported the same patient population and focused mainly on visual field data and patient age. As studies reporting routine care have shown more than 10‐fold differences in median rates of MD progression per year, Boodhna et al. (2015) chose to report the distribution of slow, medium, and fast rates of progression in their glaucoma patients.

Despite many similarities in the three countries' glaucoma guidelines, the recommendations of the latest editions of glaucoma guidelines in Sweden (Jóhannesson et al., 2024) and the United Kingdom NICE guideline (2022) also differ from the Finnish glaucoma guidelines on certain points (Heijl, 2024; Leinonen et al., 2024). For example, compared to the 1–2‐year test set interval recommended by the Finnish guideline, the Swedish guidelines (like the guidelines of the European Glaucoma Society 2021) recommend taking 5–6 visual fields during the first 2 years in newly detected glaucoma. In the UK, based on patient risks and clinical judgement, the NICE guideline (2022) recommends 2–18‐month assessment/test intervals for glaucoma and 4‐ to 24‐month intervals for ocular hypertension (OHT). Crabb et al. (2014) reported that VF monitoring in England is carried out annually on average.

Unlike the UK and Sweden, OHT patients and glaucoma suspects are not routinely followed at Tays (Sulonen et al., 2024), nor does the Finnish guideline recommend routine measurements for central corneal thickness (Leinonen et al., 2024), which also differs from the Swedish and NICE guidelines (Jóhannesson et al., 2024; National Institute for Health and Care Excellence, 2022). The NICE guideline does not include choosing wisely recommendations, while the Finnish guideline has had them since 2019 (Tuuminen et al., 2019), like the recent 2024 Swedish guidelines (Jóhannesson et al., 2024). In addition, UK optometrists (e.g., Gray et al., 2000) traditionally have had an important role in providing primary glaucoma care. Both in Finland and Sweden (Heijl et al., 2013), ophthalmology clinics provide both primary and secondary glaucoma care.

### 2.4 Analysis of Tays RWD outcomes

We analysed baseline MDs on the basis of VF damage by comparing right versus left eyes and better versus worse eyes. MDs differing less than 1 dB were randomly designated as the better or worse eye. Distributions of baseline MDs were analysed in five severity groups: (1) MD better than −2 dB, (2) MD between −2 dB and − 5.9 dB, (3) MD between −6 dB and − 11.9 dB, (4) MD between −12 dB and − 17.9 dB, and (5) MD worse than −18 dB. Further, we defined three groups of VF worsening rates: (1) slow rate less than 0.5 dB per year, (2) medium rate between 0.5 and 1.5 dB per year, and (3) fast rate more than 1.5 dB per year. In addition to using MD to define better/worse eyes at baseline, we also analysed the MD worsening distributions when defining better/worse eyes. (1) based on the mean of all MD values per eye during the follow‐up, and (2) when better and worse eyes were alternating during follow‐up.

The distribution of median ages per MD at baseline and MD worsening rates in better and worse eyes were analysed in five age groups: (1) less than 50 years, (2) between 50 and 59 years, (3) between 60 and 69 years, (4) between 70 and 79 years, and (5) 80 years or older. Similarly, the distribution of median IOPs per MD at baseline and MD worsening rates in better and worse eyes were analysed in five IOP groups: (1) less than 15 mmHg, (2) between 15 and 19 mmHg, (3) between 20 and 24 mmHg, (4) between 25 and 29 mmHg, and (5) 30 mmHg or higher.

### 2.5 Ethics and governance

This study (R21519/2021) was approved by the Tays Research Services of Tampere University Hospital and conducted in accordance with the Declaration of Helsinki. The Ethics Committee of Pirkanmaa Wellbeing Service County did not require ethics approval for the study as it evaluates aggregated unidentified real‐world data of glaucoma patients.

The use of clinical data in this study complies with the Finnish Act on the Secondary Use of Health and Social Data (552/2019), data security regulations of Tampere University Hospital, and the Finnish recommendations on research ethics. According to the Act, patients' personal data can be used without informed consent only in officially audited and secured environments under the permission granted by the organisation responsible for patient care, in our case, Tampere University Hospital. The patients' raw data cannot be transferred from the data‐secured Tays Research Cloud for statistical analyses.

The clinical data have been transferred and are stored in a secured environment based on Azure cloud technology in the Tays Research Workspace. The research group has been provided with a virtual server in its own sandbox with no direct internet connections. The authentication of each member of the research group is verified with a unique username and password. Remote access to the sandbox is done using a secure, encrypted virtual private network (VPN) connection. The service provider checks and loads both the source data and the needed software programs into the sandbox.

### 2.6 Statistical analyses

Non‐normally distributed and categorical variables were evaluated using the Wilcoxon signed‐rank test, Kruskal‐Wallis test, and chi‐square test. The level of statistical significance was set at *p* < 0.05. Statistical analyses were performed only in data sets with adequate sample size and conducted using SPSS (IBM Corp. Released 2013). IBM SPSS Statistics for Windows, Version 28.0.: IBM Corp) and the statistical R software (R Foundation for Statistical Computing).

## 3 RESULTS

### 3.1 Real‐world data at Tays Eye Centre in 2012–17

**FIGURE 1:**
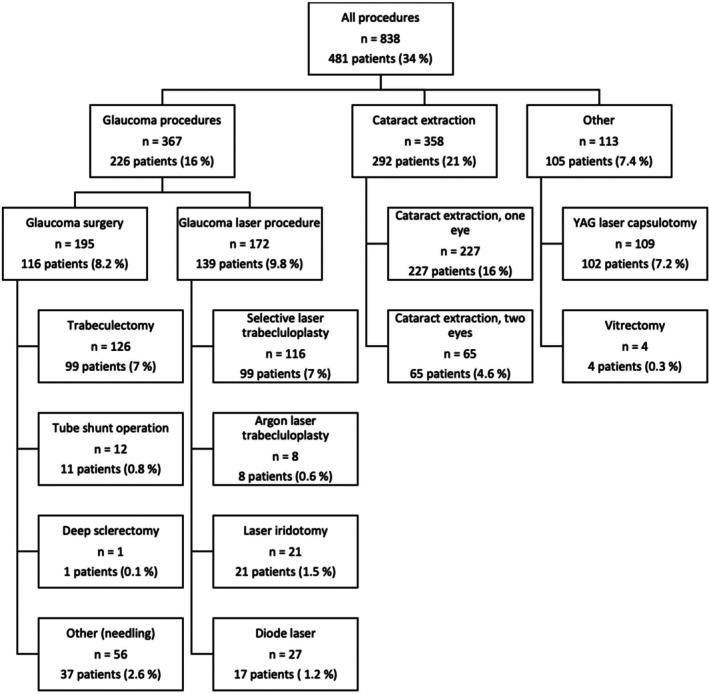
Laser and surgical procedures performed during 2012–17 in the study population of 1413 patients.

The clinical characteristics at baseline and at 5 years are presented in Table 2, and glaucoma‐related procedures in 2012–17 in Figure 1. Higher age was generally related to faster progression (Table 3, Column A, and Table S2) (*p* < 0.001, Kruskal‐Wallis test). MD worsening at rates of 0.5–1.5 dB per year tended to occur 4 years earlier in worse eyes compared to better eyes (Table S2). Both in the eyes with lower and higher IOP in 2012, mean IOPs were statistically significantly lower in 2017 (*p* < 0.001, Wilcoxon signed‐rank test, Table 2). However, when categorising eyes by baseline and worsening of MD, the median IOPs in 2012 and in 2017 were the same (14.5 mmHg in both better and worse eyes, with no statistical significance, Kruskal‐Wallis test) (Column A in Table 3 and Table S3).

**TABLE 2:** Clinical characteristics of the 1413 study patients at baseline and at 5 years.

	2012	2017
**Age (years)**		
Mean (SD, standard deviation)	68 (11)	74 (11)
Median (IQR, interquartile range)	70 (63–76)	75 (68–81)
Range	18–94	23–99
Female (%)	64	64
Patients with only single‐eye data available, *n* (%)		4 (0.3%)
**Glaucoma diagnosis type (%)**		
Ocular hypertension or glaucoma suspect		9.2
Primary open‐angle glaucoma		43
Normal tension glaucoma		18
Pseudoexfoliation glaucoma		12
Secondary glaucoma		2.4
Other or missing		7.8
Pigmentary glaucoma		1.4
Primary angle‐closure glaucoma		4.4
Mixed diagnosis between eyes		2.9
**Number of medications**		
Mean (SD)	1.5 (1.2)	1.2 (0.9)
0 medications (%)	23	25
1 medication (%)	31	38
2 medications (%)	25	30
3 medications (%)	15	6.4
4 or more (%)	5.3	1.3
**Intra‐ocular pressure (IOP), mmHg**		
Mean (95% CI) of the eye with lower initial IOP	14.6 (14.4–14.9)*	13.0 (12.8–13.2)*
Mean (95% CI) of the eye with higher initial IOP	16.5 (16.2–16.7)*	15.1 (14.9–15.4)*
**Visual acuity (VA), ETDRS letters**		
Mean (SD) of the eye with better initial VA	79 (10)	78 (12)
Mean (SD) of the eye with worse initial VA	72 (17)	70 (19)

Abbreviation: CI, confidence interval.

* *p* < 0.001, Wilcoxon signed‐rank test.

**TABLE 3:** Glaucoma real‐world data (RWD) collected in Tampere University Hospital (Tays) and six published RWD sets from Sweden and England. The items highlighted in grey are referenced in the Results and Discussion sections.

	A	B	C	D	E	F	G
Study	Current study	Heijl et al. (2013)	Kirwan et al. (2014)	Boodhna et al. (2015)	Boodhna & Crabb (2015)	Kelly et al. (2019)	Kelly et al. (2020)
Area, country	Tays, Finland	Skåne, Sweden	Portsmouth, England	Four NHS Trusts, England	Four NHS Trusts, England	Five NHS Trusts, England	Five NHS Trusts, England
Population of the serving area(s)	560 000	300 000	NR	NR	NR	NR	NR
Patients receiving care in the area/unit	88% (44% 2‐yearly)	75%	95%	NR	NR	NR	NR
Level of provided care	Primary & Secondary	Primary & Secondary	Secondary	Secondary	Secondary	Secondary	Secondary
Data collection	Manual	NR	EHR	EHR	EHR	EHR	EHR
Time period(s)	2012–2017	1996–2005	1999–2010	1999–2003 vs. 2003–2008	1999–2011	NR	2000–2015
The onset year	2012	Variable	Variable	1999 & 2003	1999–2001 & 2009–2011	Variable	Variable
Follow up with data	5 years	7.8 years	6.7 years	Fixed to 4 years	Cross‐sectional data at diagnosis	NR	Max 5 years
Data collection period	5 years	9.4 years	11 years	13 years	13 years	2000–2015	2000–2015
Total no. patients in onset or last year	4121 in 2012	NR	NR	88 954 in 2012	83 573 in 2012	73 944 in 2015	73 944 in 2015
Selection criteria							
Follow‐up	5‐year f‐up	≥5‐year f‐up	IQR 5 years	Fixed 4‐year f‐up windows	Cross‐sectional data at diagnosis	≥4‐year f‐up	Max 5 years
Number of VFs	≥ 2 VFs	≥5 VFs	>5 VFs	≥ 4 VFs (first removed)	First 2VFs in 1999–2001 & 2009–11	≥4 VFs (first removed)	≥ 3 VFs
Age	> 18 years	>30 years	NR	> 40‐year olds	NR	NR	NR
Diagnosis	All dg included	Abnormal GHT	All dg included	All dg included	MD outside normal limits twice	MD outside normal limits twice	9624/73 994 = 13% with OHT
Exclusion criteria	False positives ≥15%	EMGT pts., blindness	NR	NR	NR	False positives ≥15%	20% VF excl. (FP > 15%, fix losses > 20%)
							VA <0.3, cataract, corneal pathology
Number of follow‐up visual fields per patient	4.5 (mean)	8.9 (mean)	6 (median)	5 (median), 4–5	NA (cross‐sectional data)	8	≥ 3 VFs
Age, years (median) at onset year	70 years	71 (mean)	67 years	65 years & 66 years	66 years	71 years	60 years
Female	64%	63%	NR	NR	NR	53%	54%
Diagnoses	All dg included	POAG 62%, PEX 38%	All dg included	All dg included	Newly diagnosed glaucoma	Glaucoma	OHT (70% treated)
Number of patients fulfilling the criteria	1413	583	2208	13 984	25 521	19 264	3153
Proportion of all pts	34%	NR	NR 16%	31%	26%	33% of OHTs	
Better and/or worse eyes analysed	Better & worse eyes	Worse eyes only	Better eyes only	Better/worse not defined	Worse eyes only	Worse eyes only	Higher IOP, more ‘serious’ label
Baseline MD (median), all eyes	−2.9 dB	NR	−0.9 dB	−2.9 dB & −2.6 DB	MD > −6 dB 1999 vs. 2011 41% – >50%	NR	Worse eyes: −0.45 dB
Better eyes	−2.0 dB	NR	−2.0 dB	NR	MD < −12 dB 1999 vs. 2011 30% – >21%	Worse eyes 24% (23–30%)	NR
Worse eyes	−4.3 dB	Worse eyes: −10 dB	‐ 3.2 dB	NR	NR	NR	NR
Rate of worsening/year (median)	Better eyes: 0.06 dB/year	NR	Better eyes 0.15 dB/year	All eyes 0.11 dB/year & 0.06 dB/year	NA (cross‐sectional data)	NR	Rate of worsening not reported
	Worse eyes 0.22 dB/year	Worse eyes 0.62 dB/year	NR	25% at 0.53 dB/year & 0.51 dB/year		Worse eyes 0.21 dB/year	Overall 18% with progression
		NR	NR	10% at 1.14 dB/year & 1.13 dB/year		NR	14% of treated/27% of untreated
% of MD worsening/year							
Less than 0.5 dB	Better 79%/Worse 75%	NR	79%	74% & 75%	NA (cross‐sectional data)	NR	90% mild to moderate loss
Less than 0.3 dB	NR	NR	66%	NR		NR	10% advanced loss using Glaucoma Staging System (GSS 2) (Brusini & Filacorda, 2006)
0.3–1 dB	NR	NR	23%	NR		NR	
0.5–1.5 dB	Better 16%/Worse 18%	NR	NR	20% & 19%		NR	
1–2 dB	NR	NR	7%	NR		NR	
More than 1.5 dB	Better 5%/Worse 7%	NR	NR	6% & 6%		NR	VF defect in 6 stages and in 3 types based MD and corrected Pattern Standard Deviation (CPSD)
More than 2 dB	NR	NR	2%	NR		NR	
More than 2.5 dB	0.1% (two worse eyes)	6%	NR	NR		NR	
IOP, mmHg (median)							
Initial visit	Better 14.5 mmHg/Worse 14.5 mmHg	20 mmHg (mean)	NR	NR	NR	NR	24 mmHg (median)
Last visit	Better 14.5 mmHg/Worse 14.5 mmHg	18 mmHg (mean)	NR	NR	NR	NR	21 mmHg (median)
Laser therapy	10%	22%	NR	NR	NR	NR	Proportion not reported
Glaucoma surgery	8%	10%	NR	NR	NR	NR	Proportion not reported
Factors related to faster progression							
Age	+	+	NR	+	Baseline MD 0.01 dB worse per ageing year	NR	+
IOP	**−**	+	NR	NR	NR	NR	**−**
PEX	NR	**−**	NR	NR	NR	NR	NR
Baseline MD	+	NR	NR	Slow and medium rate NS	NR	NR	NR
	Worst MD, slower rate	Worst MD, slower rate	NR	Worst MD, slower rate	NR	NR	NR

The median MD in 6347 VFs of left eyes was −4 dB (interquartile range, IQR −8, −1) compared to the median MD of −3 dB in 6295 VFs in right eyes (IQR –8, −1) (*p* = 0.06 Wilcoxon signed‐rank test). The better/worse eye distribution was 55%/45% in right eyes and 47%/53% in left eyes (*p* < 0.001, Chi‐squared test).

Column A in Table 3 and Table S4 describes the distributions and the numbers of eyes at Tays in different MD worsening rate categories in the better and worse eyes per baseline MD groups (not significant). The distributions were similar regardless of the definition of the better/worse eyes (data not shown).

The mean overall cost per patient was 312 € per year in 2012–17, that is, 321 €, 325 €, 316 €, 289 €, 307 €, and 312 €, respectively. The number of glaucoma patients per year visiting Tays increased 25% during that time (from 4121 to 5154).

### 3.2 Comparison of real‐world data sets

Table 3 presents summary data of this study (Column A) and the published data from the Swedish study (Column B) and the five studies from England (Columns C‐G). The main message of Table 3 is that it demonstrates large inter‐site variability in reporting in most parameters, also in studies published in England.

Although this variability makes it difficult to draw meaningful conclusions to support decision‐making, a few overall observations draw attention specifically related to the highest risk groups. Highlighted in columns A and D in Table 3, during three different time periods (1999–2003 and 2003–2008 in England as well as at Tays in 2012–17), the proportions of the medium and fast MD worsening rates were very similar, i.e., medium rate progression was 16–18% at Tays vs. 19–20% in England, and fast rate progression was 5–7% at Tays vs. 6% in England. Year 2003 in England was chosen as a landmark because the frequency of prostaglandin use overtook beta blocker monotherapy that year (Boodhna et al., 2015). During a bit longer period in 1999–2011 in England (column E), the proportion of newly detected early glaucoma cases increased (from 41% to 50%), and the proportion of advanced cases decreased (from 31% to 20%). However, during almost the same time periods in column D (1999–2003 and 2003–2008), the percentages of eyes showing medium and fast rates of VF progression were the same. In addition, four studies (columns A, B, D, and G) reported age being a risk factor for progression.In three studies (columns A, B, and D) the progression rates slowed in advanced glaucoma due to truncation (floor effect).

## 4 DISCUSSION

The need to improve information gathering and knowledge of the work being done in health care has been recognised for decades (Yates, 1987), likewise studies reporting that more care and more spending as such have not led to better access to care, nor to better quality, outcomes, or satisfaction with care (Fisher et al., 2003a and Fisher et al., 2003b). However, the literature about benchmarking of glaucoma outcomes using routine comprehensive RWD is scarce but is expected to rapidly increase with the implementation of structured EHRs. Simultaneously, the high variability observed in terminology relating to evaluation methodologies, such as audit, performance review, and benchmarking, calls for agreement. In general, e.g., the Public Risk Management Association (PRIMA, 2024) summarises that although these three methods differ from each other, they are also tied together, and it is very difficult to perform these evaluations without simultaneously evaluating the financial information as well.

In brief, for example, audits are typically undertaken by independent actors to evaluate performance against pre‐determined standards (PRIMA,^2024^), such as adherence to guidelines. For example, ophthalmologists' adherence to glaucoma test sets has been reported, e.g., in Spain, Sweden, and the UK (Batra et al., 2018; Castejón‐Cervero et al., 2011; Lindén et al., 2013), and in Australia by optometrists (Toomey et al., 2024). Benchmarking, on the other hand, serves the important comparative purpose of evaluating the impact of different organisations' performance on health outcomes (Hart et al., 2009; Joint Commission Resources Inc., 2012). The principles of benchmarking also apply to the evaluation of real‐world eye care; i.e., cost‐effectiveness can only be shown in relation to a defined alternative, such as care practices of different organisations. Nothing is intrinsically cost‐effective (Tuulonen, 2011; Tuulonen et al., 2022).

The motivators for developing benchmarking capabilities relate to improving quality as well as fighting inequalities and unwarranted variability in care delivery, accountability, and transparency of care, as well as responding to rising demand versus limited resources (Thonon et al., 2015; Tuulonen et al., 2022). Benchmarking models can be developed, e.g., from existing and published data, from consensus statements, from national and/or international standards, etc. (Toomey et al., 2024). The metrics need to be (1) meaningful both for patients and clinicians, (2) easily and systematically recordable and available from EHRs, and (3) understandable to aid decision‐making at different levels of health care. In addition, it is crucial to confirm that we measure, report, and compare the same aspects of care using the same metrics and methods (Toomey et al., 2024).

Adapting trial designs on the basis of real‐world evidence has a growing role in clinical, regulatory, and payer decision‐making and requires collaboration of clinicians with deep practice experience and questioning minds working closely together (Dreyer & Mack, 2023; Liu et al., 2021). In the Finnish *aces‐rwm* cost‐effectiveness ecosystem, clinical metrics for disease‐specific bespoke EHR tools have been agreed upon within the ‘Big Four’ working groups representing all Finnish university eye clinics (Table 1). Similarly, the *aces‐rwm* ecosystem has published its overall RWD evaluation framework, including cost‐effectiveness (Tuulonen et al., 2022). Therefore, the bespoke *aces‐rwm* IT‐tool package developed in 2019–23 already includes a technical platform for benchmarking. This study represents the first step in the process for developing a master protocol to produce outcomes for benchmarking glaucoma care, to be used in the *aces‐rwm* benchmarking platform, enabling comparison of outcomes between healthcare delivery units nationally and internationally.

The *aces‐rwm* benchmarking platform is planned to work as a searchlight for scanning the similarly structured RWD data sets between clinics in peer‐to‐peer benchmarking. Both detected differences and similarities in data sets and outcomes may be valuable and guide a deeper understanding of care practices, including their impacts on outcomes. Because the Finnish Act on Secondary Use of Health Data prevents comparison of raw RWD between healthcare units, analyses of outcomes are primarily based upon distributions, such as in different stages and progression rates in glaucoma. This approach has also been used in England but with different classifications (Table 3, Columns C–D). In Finland, additional statistical analyses of patient‐specific raw data in each unit can only be done within the secured data environment of each hospital.

Table 3, comparing just a few RWD studies in three countries, clearly demonstrates large variability in reporting and calls for developing a master protocol. Table 3 is neither able to help nor to provide pragmatic guidance to the essential questions, such as ‘should the glaucoma clinic in Tays Eye Centre change its daily care practices to ensure better well‐being for patients with available resources?’. Regarding visual disability and overall resource allocation, obviously all ‘Big Four’ eye diseases need to be considered in such decision‐making, which requires the development of a master protocol for each ‘Big Four’ disease (Tuulonen et al., 2022). For example, at Tays the number of glaucoma visits increased 25% between 2012 and 2017 while the number of AMD injections more than doubled during the same period; however, without doubling the overall resources (Tuulonen et al., 2022). Determining what matters most in improving eye care requires deeper understanding and further development of the care processes in all ‘Big Four’ eye diseases. Considering the increasing number of EHR‐RWD sets, continuing the variable reporting and creating summary tables like Table 3 is not justifiable and exposes such work to inadvertent misinterpretations of the published data sets.

Interestingly, despite the large variability in reporting shown in Table 3, as well as the differences in guidelines and eye care structures between Tays and England, we see distinct similarities in the proportions of medium and fast progression rates in three different time periods, i.e., 1999–2003 and 2003–2008 in England, and 2012–17 at Tays. Likewise, approximately 10% of patients registered as sight impaired have lost vision due to glaucoma in both England and Finland (Boodhna & Crabb, 2015; Tolkkinen, 2022). Also, when comparing cross‐sectionally, 2019 EHR‐RWD at Tays (Sulonen et al., 2024) and in England in 2016 (Fu et al., 2023), there were only a few differences, e.g., more females in Tays (62% vs. 52% in England; similar differences also in Table 3), more laser procedures in Tays (14% vs. 1% in England), and smaller proportions of OHT and glaucoma suspects at Tays (28% vs. 41% in England). Regarding the latter, both Kelly et al. (2019) and Fu et al. (2023) specifically suggested that many patients in England may require less intensive follow‐up and that glaucoma care seems to be disproportionately directed toward patients with mild and low‐risk glaucoma. In addition, a questionnaire revealed that many glaucoma specialists in England considered their recommendations for VF testing frequency impractical with current resources (Crabb et al., 2014).

The overall aim of the *aces‐rwm* ecosystem is not only to benchmark the outcomes in Finnish university eye clinics with each other but also obviously, with clinics in other countries to figure out what would represent ‘good enough’ care practice for the all ‘Big Four’ eye diseases in relation to available resources (Table 1). Among the five English glaucoma studies in Table 3, only one (Kelly et al., 2019) compared the data sets between the five centres with over 8‐fold differences in their patient volumes. Although the authors reported the differences as generally being minor, the patients in the smallest centre were the oldest (mean age 75 years vs. 69–71 years in the other four centres), had the largest proportion of advanced VF loss at presentation (30% vs. 23–25% in the other centres), and showed the fastest progression rate (−0.37 dB/year vs. between −0.10 and −0.26 dB/year at 4 other centres). Thus, benchmarking focused the light on this difference, but the finding as such cannot determine what might represent ‘better’ or ‘worse’ care, or which clinic would benefit from changing its care practices. The reasons for the differences were not discussed by Kelly et al. (2019). Instead of simply concluding that the results indicating the smallest clinic being ‘worse’, it is also possible that this clinic may have agreed with optometrists to refer only the highest‐risk patients to secondary care, i.e., the exact policy suggested by the authors (Kelly et al., 2019) and Fu et al. (2023) in England.

As the volume of EHR‐RWD is huge and constantly increasing, based on the RWD analyses in this study and as well as a 2019 EHR‐RWD at Tays (Sulonen et al., 2024), we present in Table 4 the principles of how the automatically produced *aces‐rwm* master protocol could best serve benchmarking purposes. This master protocol is a prototype and requires further refinement with the help of RWD from other national and international clinics. In any case, the platform will include all recorded structured data and will also have filters for selecting subgroups, e.g., different ages, gender, diagnosis, treatments, follow‐up periods, etc., depending on the clinical or study question of interest. The platform is planned to update the data in real‐time, based upon the filter selections. This will allow the benchmarking units to compare their clinical data sets and to work online to detect similarities and differences, which will guide further evaluation of care protocols and enable continuous follow‐up of real‐world outcomes, choose meaningful questions, study designs, etc.

**TABLE 4:** Principles of the automatically produced master protocol on the aces‐rwm benchmarking platform to be further developed in the next stage of the development process. All data are reported per year.

**Serving area**
Population
Number of patients using reimbursed glaucoma medications: Total/New/Deceased/Net increase of users
% of glaucoma medication users receiving care in the clinic
**Care**
Primary / Secondary / Tertiary
**Evaluation**
Selection criteria: Time period / Minimum follow‐up / Number of follow‐up visual fields, etc.
Exclusion criteria
**Proportion (%) of patients fulfilling the selection criteria**
Per all glaucoma patients in the clinic
**Age** (years) mean/median
Distribution (%): <50 years / 50–59 years / 60–69 years / 70–79 years / ≥ 80 years
**Gender**
Distribution (%): Female / Male / Other
**History**
Risk factors: Distribution of positive family history, exfoliation, etc.
**Diagnosis** Distribution (%): ICD10 diagnosis H40 / Other ophthalmic diagnoses
**Eye**
Better / Worse eye: MD difference ≥1 dB, random eye for MD within + − 1 dB between eyes
Right / Left eye
**Test results**
*VA (EDTRS) Mean / Median*
Distribution (%): Snellen <0.3 / 0.3–0.5 / >0.5
Refraction
*IOP (mmHg) Mean/median*
Distribution (%): <15 mmHg / 15–19 mmHg / 20–24 mmHg / 25–29 mmHg / ≥30 mmHg
**Clinical assessment**
*ONH / RNFL / VF*
Distribution (%): Normal / Glaucoma / Other / Cannot be assessed / Not tested
*Distribution (%) of abnormality in ONH / RNFL / VF*
≥2 out 3 abnormal / 1 out of 3 abnormal / 0 out of 3 abnormal / IOP ≥30 mmHg
ONH haemorrhage: Yes / No
**Clinical assessment of progression**
Distribution (%)
Yes / No / Cannot be assessed / No prior tests
**MD** (dB)
Distribution (%): > −2 dB / −2 → −6 dB / −6 → −12 dB / −12 → 18 dB / <−18 dB
**Proportion of MD worsening rates per year**
Distribution (%): <0.5 dB / 0.5–1.5 dB / >1.5 dB
**Treatment distribution (%)**
Medication / Laser / Surgery
Prostaglandins / Beta blockers / Carbonic anhydrase inhibitors / Alpha agonists / Other
SLT / Laser iridotomy / Other
Trabeculectomy / Tube / MIGS / Other
**Quality of life**
Total score / Vision score
Proportion of responders (%)
**Cost per patient per year (€)**
Costs of visits, tests, and procedures divided by the number of patients

The US National Institutes of Health (2024) defines a master protocol as a trial design that can test multiple subpopulations in parallel under a single protocol, without the need to develop new protocols for every trial. This is the exact aim of the *aces‐rwm* ecosystem, i.e., to develop a master protocol for each of the ‘Big Four’ eye diseases. The glaucoma master protocol includes not only VF data but also all clinical EHR‐RWD, structural evaluations of ONH and RNFL and their progression, as well as different treatments. In the next step, the prototype master protocol (Table 5) will be further refined by comparing the Tays 2012–17 RWD of this study to the Tays structured 2019–23 EHR RWD (including also health‐related quality of life measures) and by comparing the Tays 2019 RWD and Umeå (Sweden) data to each other.

**TABLE 5:** Example of how the *aces‐rwm* benchmarking platform is planned to work. E.g., if two clinics would be interested in comparing their 5‐year outcomes of exfoliative eyes receiving laser trabeculoplasty treatment. After making the same selections in an online meeting, the benchmarking platform produces real‐time, similarly structured reports for both clinics. In case wishing to modify selections, the platform updates each report in real time and enables saving all comparison reports for later evaluation, planning a study, etc.

**Serving area**	
Population	
Number of patients using reimbursed glaucoma medications	Total / New / Deceased
% of glaucoma medication users receiving care in the clinic	
**Care**	
Primary / Secondary / Tertiary	
**Selected time period**	2019–23
	**Baseline selections in 2019**
**Eye with better/worse MD**	Worse (≥1 dB)
	Random eye for MD + −1 dB
**History**	
Risk factors (family history, exfoliation, etc.)	Exfoliation+
Highest untreated IOP	≥22 mmHg
**Diagnosis (ICD10)**	H40.X
Other diagnosis (ICD10)	No exclusions
**Test results**	
VA	≥0.5 Snellen
Refraction / IOP	No exclusions
**Clinical assessments**	
ONH / RNFL‐/‐VF	≥1 out 3 glaucomatous in 2019
Normal /Glaucoma / Other / Cannot be assessed / Not tested	Reported
ONH haemorrhage	No exclusions
**MD at baseline**	2019
>−2 / −2 → −6 / −6 → −12 / −12 → 18 /<−18 dB	Worse than −2 dB
Proportion of selection	%
**Proportion of worsening rate per year in 2019–23**	
Less than 0.5 / 0.5–1.5 dB / More than 1.5 dB	Reported
**Clinical assessments of progression**	
Yes / No / Cannot be assessed / No prior tests	Reported
**Treatment**	
Medication / Laser / Surgery	SLT
**Quality of life**	
Total score / Vision score	Reported
Proportion of responders %	Reported
**Cost per patient per year (€)**	
Visits, tests, and procedures divided by the number of patients	Reported

## AUTHOR CONTRIBUTIONS

All authors meet all four criteria for authorship by the International Committee of Medical Journal Editors (ICMJE).

## CONFLICT OF INTEREST STATEMENT

PH: Advisory board honorarium from Novartis and congress travel fees from AbbVie. AT, KV, EL: (none). SL: Lecture fees from Santen, Thea, and AbbVie. GJ: Lecture and consultancy fees from AbbVie, Santen, and Thea. AV: Lecture and consultancy fees from Santen. HUJ: Advisory board member of AbbVie, Bayer, Novartis, and Roche; lecture fees from Santen and Thea.

## Supporting information


Data S1:

Data S2:

Data S3:

Data S4:

## References

[aos17453-bib-0001] Batra, R. , Sharma, H.E. , Elaraoud, I. & Mohamed, S. (2018) Resource planning in glaucoma: a tool to evaluate glaucoma service capacity. Seminars in Ophthalmology, 33, 733–738. 29283293 10.1080/08820538.2017.1418012

[aos17453-bib-0002] Boodhna, T. & Crabb, D.P. (2015) Disease severity in newly diagnosed glaucoma patients with visual field loss: trends from more than a decade of data. Ophthalmic & Physiological Optics, 35, 225–230. 25545852 10.1111/opo.12187

[aos17453-bib-0003] Boodhna, T. , Saunders, L.J. & Crabb, D.P. (2015) Are rates of vision loss in patients in English glaucoma clinics slowing down over time? Trends from a decade of data. Eye (London, England), 29, 1613–1619. 26315701 10.1038/eye.2015.161 PMC5129813

[aos17453-bib-0004] Bousquet, J. , Illario, M. , Farrell, J. , Batey, N. , Carriazo, A.M. & Zurkuhlen, A.J. (2019) The reference site collaborative network of the European innovation partnership on active and healthy ageing. Translation Medicine University of Salerno, 6(19), 66–81. PMC6581486 31360670

[aos17453-bib-0005] Brusini, P. & Filacorda, S. (2006) Enhanced glaucoma staging system (GSS 2) for classifying functional damage in glaucoma. Journal of Glaucoma, 15, 40–46. 16378017 10.1097/01.ijg.0000195932.48288.97

[aos17453-bib-0006] Castejón‐Cervero, M.A. , Jiménez‐Parras, R. , Fernandez‐Arias, I. , Teus‐Guezala, M.A. & IMCA Study Group . (2011) Evaluation of compliance with the EGS guidelines in Spain, using achievable benchmarks of care (ABC®) methodology: the IMCA study. European Journal of Ophthalmology, 21, 149–155. 21058273 10.5301/ejo.2010.5973

[aos17453-bib-0007] Crabb, D.P. , Russell, R.A. , Malik, R. , Anand, N. , Baker, H. , Boodhna, T. et al. (2014) frequency of visual field testing when monitoring patients newly diagnosed with glaucoma: mixed methods and modelling. Health Services and Delivery Research, 2(27), 1–102. Available from: 10.3310/hsdr02270 25642569

[aos17453-bib-0008] Dreyer, N.A. & Mack, C.D. (2023) Tactical considerations for designing real‐world studies: fit‐for‐purpose designs that bridge research and practice. Pragmatics & Cognition, 14, 101–110. 10.2147/POR.S396024 PMC10541678 37786592

[aos17453-bib-0009] Fisher, E.S. , Wennberg, D.E. , Stukel, T.A. , Gottlieb, D.J. , Lucas, F.L. & Pinder, E.L. (2003a) The implications of regional variations in Medicare spending. Part 1: the content, quality, and accessibility of care. Annals of Internal Medicine, 138, 273–287. 12585825 10.7326/0003-4819-138-4-200302180-00006

[aos17453-bib-0010] Fisher, E.S. , Wennberg, D.E. , Stukel, T.A. , Gottlieb, D.J. , Lucas, F.L. & Pinder, E.L. (2003b) The implications of regional variations in Medicare spending. Part 2: health outcomes and satisfaction with care. Annals of Internal Medicine, 138, 288–298. 12585826 10.7326/0003-4819-138-4-200302180-00007

[aos17453-bib-0011] Franklin, J.B. , Marra, C. , Abebe, K.Z. , Butte, A.J. , Cook, D.J. , Esserman, L. et al. (2024) Modernizing the data infrastructure for clinical research to meet evolving demands for evidence. Journal of the American Medical Association. 10.1001/jama.2024.0268. 39102333

[aos17453-bib-0012] Fu, D.J. , Ademisoye, E. , Shih, V. , McNaught, A.I. & Khawaja, A.P. (2023) Burden of glaucoma in the United Kingdom: a multicenter analysis of United Kingdom glaucoma services. Ophthalmol Glaucoma, 6, 106–115. 35973529 10.1016/j.ogla.2022.08.007

[aos17453-bib-0013] Gray, S.F. , Spry, P.G. , Brookes, S.T. , Peters, T.J. , Spencer, I.C. , Baker, I.A. et al. (2000) The Bristol shared care glaucoma study: outcome at follow up at 2 years. The British Journal of Ophthalmology, 84, 456–463. 10781507 10.1136/bjo.84.5.456 PMC1723467

[aos17453-bib-0014] Gregori, N.Z. , Feuer, W. & Rosenfeld, P.J. (2010) Novel method for analyzing Snellen visual acuity measurements. Retina, 30, 1046–1050. 20559157 10.1097/IAE.0b013e3181d87e04

[aos17453-bib-0015] Hart, A. , Northmore, S. & Gerhardt, C. (2009) Briefing Paper: Auditing, Benchmarking and Evaluating Public Engagement. National Co‐ordinating Centre for Public Engagement Watershed Media Centre, Research Councils UK and the Welcome Trust, January 2009. https://talloiresnetwork.tufts.edu/wp‐content/uploads/AuditingBenchmarkingandEvaluatingPublicEngagement.pdf (accessed September 21, 2024).

[aos17453-bib-0016] Heijl, A. (2024) New Finnish and Swedish glaucoma guidelines. Acta Ophthalmologica, 102, 133–134. 38084645 10.1111/aos.16600

[aos17453-bib-0017] Heijl, A. , Buchholz, P. , Norrgren, G. & Bengtsson, B. (2013) Rates of visual field progression in clinical glaucoma care. Acta Ophthalmologica, 91, 406–412. 23066646 10.1111/j.1755-3768.2012.02492.x PMC3798127

[aos17453-bib-0018] Higgins, B.E. , Leonard‐Hawkhead, B. & Azuara‐Blanco, A. (2024) Quality of reporting electronic health record data in glaucoma: a systematic literature review. Ophthalmology Glaucoma, 7, 422–430. 38599318 10.1016/j.ogla.2024.04.002

[aos17453-bib-0019] Jóhannesson, G. , Stille, U. , Taube, A.B. , Karlsson, M. , Kalaboukhova, L. , Bergström, A. et al. (2024) Guidelines for the management of open‐angle glaucoma: National Program Area eye Diseases, National Working Group Glaucoma. Acta Ophthalmologica, 102, 135–150. 38164112 10.1111/aos.16599

[aos17453-bib-0020] Joint Commission Resources Inc . (2012) Benchmarking in health care.

[aos17453-bib-0021] Kelly, S.R. , Bryan, S.R. , Sparrow, J.M. & Crabb, D.P. (2019) Auditing service delivery in glaucoma clinics using visual field records: a feasibility study. BMJ Open Ophthalmology, 5(4), e000352. 10.1136/bmjophth-2019-000352 PMC6711463 31523719

[aos17453-bib-0022] Kelly, S.R. , Khawaja, A.P. , Bryan, S.R. , Azuara‐Blanco, A. , Sparrow, J.M. & Crabb, D.P. (2020) Progression from ocular hypertension to visual field loss in the English Hospital Eye Service. British Journal of Ophthalmology, 104, 1406–1411. 32217541 10.1136/bjophthalmol-2019-315052

[aos17453-bib-0023] Kirwan, J.F. , Hustler, A. , Bobat, H. , Toms, L. , Crabb, D.P. & McNaught, A.I. (2014) Portsmouth visual field database: an audit of glaucoma progression. Eye (London, England), 28, 974–979. 24875227 10.1038/eye.2013.294 PMC4135248

[aos17453-bib-0024] Kokkinen, L. & Lehto, J. (2011) Changing health care from inside out: policy entrepreneur questioning ophthalmology service production in Finland. International Journal of Public and Private Healthcare Management and Economics, 1, 16–27.

[aos17453-bib-0025] Kontiola, A.I. , Goldblum, D. , Mittag, T. & Danias, J. (2001) The induction/impact tonometer: a new instrument to measure intraocular pressure in the rat. Experimental Eye Research, 73, 781–785. 11846509 10.1006/exer.2001.1088

[aos17453-bib-0026] Kotecha, D. , Asselbergs, F.W. , Achenbach, S. , Anker, S.D. , Atar, D. , Baigent, C. et al. (2022) CODE‐EHR best‐practice framework for the use of structured electronic health‐care records in clinical research. Lancet Digital Health, 4, e757–e764. 36050271 10.1016/S2589-7500(22)00151-0

[aos17453-bib-0027] Leinonen, S. , Harju, M. , Hagman, J. , Honkamo, M. , Marttila, L. , Määttä, M. et al. (2024) The Finnish current care guideline for open‐angle glaucoma. Acta Ophthalmologica, 102, 151–171. 38174651 10.1111/aos.16612

[aos17453-bib-0028] Lindén, C. , Bengtsson, B. , Alm, A. , Calissendorff, B. , Eckerlund, I. & Heijl, A. (2013) Glaucoma management in Sweden—results from a nationwide survey. Acta Ophthalmologica, 91, 20–24. 22011061 10.1111/j.1755-3768.2011.02273.x PMC3579229

[aos17453-bib-0029] Liu, M. , Li, Q. , Lin, J. , Lin, Y. & Hoffman, E. (2021) Innovative trial designs and analyses for vaccine clinical development. Contemporary Clinical Trials, 100, 106225. 33227451 10.1016/j.cct.2020.106225 PMC7834363

[aos17453-bib-0030] National Institute for Health and Care Excellence . 2022 https://www.nice.org.uk/guidance/ng81/resources/glaucoma‐diagnosis‐and‐management‐pdf‐1837689655237 (accessed October 19, 2024).

[aos17453-bib-0031] National Institutes of Health . 2024 https://toolkit.ncats.nih.gov/glossary/master‐protocol/ (accessed September 21, 2024).

[aos17453-bib-0032] Public Risk Management Association (PRIMA) . 2024 https://primacentral.org/education/podcasts‐blog/audits‐performance‐reviews‐benchmarking/ (accessed September 21, 2024).

[aos17453-bib-0033] Social Insurance Institution of Finland . 2024 https://tietotarjotin.fi (accessed October 19, 2024).

[aos17453-bib-0034] European Glaucoma Society Terminology and Guidelines for Glaucoma. British Journal of Ophthalmology, 105(Suppl 1), 1–169. 10.1136/bjophthalmol-2021-egsguidelines 34675001

[aos17453-bib-0035] Sulonen, S. , Leinonen, S. , Lehtonen, E. , Hujanen, P. , Vaajanen, A. , Syvänen, U. et al. (2024) A prototype protocol for evaluating the real‐world data set using a structured electronic health record in glaucoma. Acta Ophthalmologica, 102, 216–227. 37753831 10.1111/aos.15763

[aos17453-bib-0036] Thonon, F. , Watson, J. & Saghatchian, M. (2015) Benchmarking facilities providing care: an international overview of initiatives. Sage Open Medicine, 3, 205. 10.1177/2050312115601692 PMC4712789 26770800

[aos17453-bib-0037] Tolkkinen, L. (2022) The Finnish Register of Visual Impairment Annual Statistics. https://cms.nkl.fi/sites/default/files/2023‐12/VALMIS%20Annual%20Statistics%202022.pdf?_ga=2.198118060.260608361.1718903236‐1365678351.1718903236 (accessed June 13, 2024).

[aos17453-bib-0038] Toomey, M. , Gyawali, R. , Ho, K.C. , Stapleton, F. , Keay, L. & Jalbert, I. (2024) Developing realistic benchmarks for glaucoma care delivery. Clinical & Experimental Optometry, 107, 196–203. 37952255 10.1080/08164622.2023.2275748

[aos17453-bib-0039] Tuulonen, A. (2011) Cost‐effectiveness of screening for open angle glaucoma in developed countries. Indian Journal of Ophthalmology, 59 Suppl(Suppl1), S24–S30. 21150030 10.4103/0301-4738.73684 PMC3038514

[aos17453-bib-0040] Tuulonen, A. , Airaksinen, P.J. , Erola, E. , Forsman, E. , Friberg, K. , Kaila, M. et al. (2003) The Finnish evidence‐based guideline for open‐angle glaucoma. Acta Ophthalmologica Scandinavica, 81, 3–18. 12631014 10.1034/j.1600-0420.2003.00021.x

[aos17453-bib-0041] Tuulonen, A. , Kataja, M. , Aaltonen, V. , Kinnunen, K. , Moilanen, J. , Saarela, V. et al. (2022) A comprehensive model for measuring real‐life cost‐effectiveness in eyecare: automation in care and evaluation of system (aces‐rwm™). Acta Ophthalmologica, 100, e833–e840. 34263537 10.1111/aos.14959

[aos17453-bib-0042] Tuulonen, A. , Kataja, M. , Syvänen, U. , Miettunen, S. & Uusitalo, H. (2016) Right services to right patients at right time in right setting in Tays Eye Centre. Acta Ophthalmologica, 94, 730–735. 27422769 10.1111/aos.13168

[aos17453-bib-0043] Tuulonen, A. , Salminen, H. , Linna, M. & Perkola, M. (2009) The need and total cost of Finnish Eyecare Services: a simulation model for 2005–2040. Acta Ophthalmologica, 87, 820–829. 19740130 10.1111/j.1755-3768.2009.01532.x

[aos17453-bib-0044] Tuuminen, R. , Sipilä, R. , Komulainen, J. , Saarela, V. , Kaarniranta, K. & Tuulonen, A. (2019) The first ophthalmic choosing wisely recommendations in Finland for glaucoma and wet age‐related macular degeneration. Acta Ophthalmologica, 97, e808. 10.1111/aos.14031 30659781

[aos17453-bib-0045] Yates, J. (1987) Why are we waiting? An analysis of hospital waiting lists. Oxford: Oxford University Press.

