# Supplementary material for: Prototype master protocol for benchmarking of real‐world follow‐up data in glaucoma
Source: Acta Ophthalmol. 2025 Feb 13;103(5):539–51. doi: 10.1111/aos.17453 (PMC12235677; doi:10.1111/aos.17453)

Supplementary table 2. Distributions of median ages per worsening rates in better and worse eyes at baseline. Higher age was related to faster VF worsening rates (p < 0.001, Kruskal-Wallis test). Worsening rates > 0.5 dB per year in the worse eyes tended to be detected at younger age compared to better eyes.


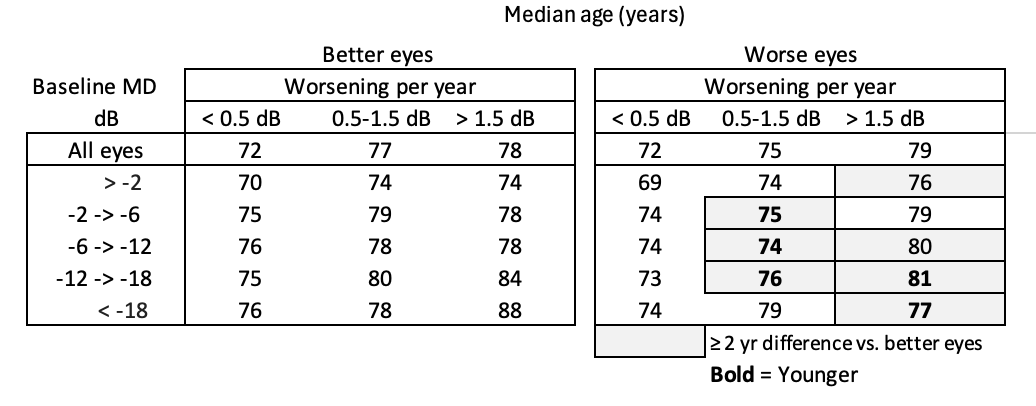

Supplement: Supplementary file 2 — Data S2: [file AOS-103-539-s003.docx]
