# Supplementary material for: Prototype master protocol for benchmarking of real‐world follow‐up data in glaucoma
Source: Acta Ophthalmol. 2025 Feb 13;103(5):539–51. doi: 10.1111/aos.17453 (PMC12235677; doi:10.1111/aos.17453)

Supplementary table 3. Median intraocular pressure (IOP) at the baseline and at 5 years as well as the mean change in IOP stratified by baseline visual field (VF) mean deviation (MD) and rate of VF worsening showed no statistically significant differences (*one eye) (Kruskal-Wallis test). For number and distribution of cases, see Table S4.


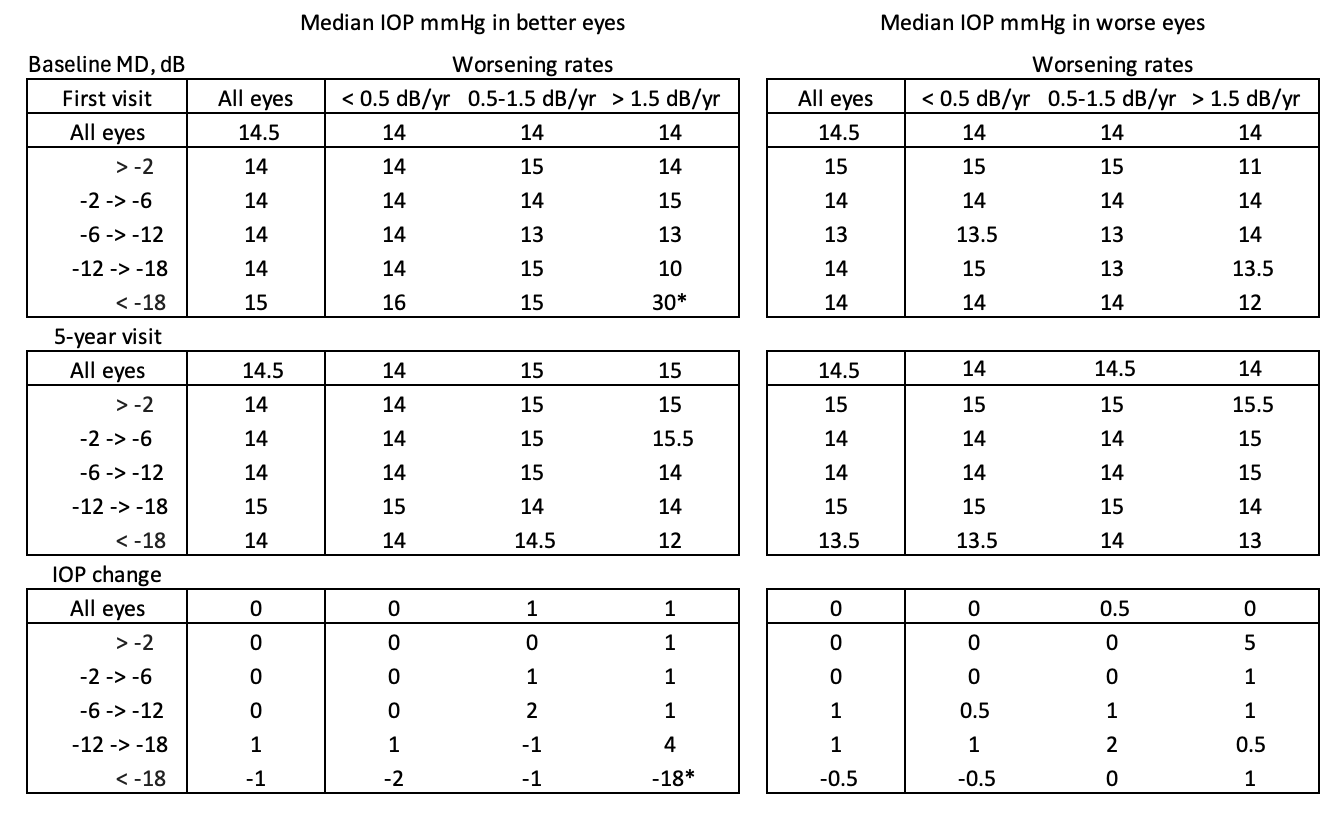

Supplement: Supplementary file 3 — Data S3: [file AOS-103-539-s002.docx]
