# Supplementary material for: Prototype master protocol for benchmarking of real‐world follow‐up data in glaucoma
Source: Acta Ophthalmol. 2025 Feb 13;103(5):539–51. doi: 10.1111/aos.17453 (PMC12235677; doi:10.1111/aos.17453)

Supplementary table 4. Worsening per year in different baseline MD groups: a) distributions between better and worse eyes (not statistically significantly different, Kruskal-Wallis test), and b) the number of eyes. In 14 better eyes (1 %), the MD was -18 dB or worse at baseline and in 66 better eyes (5 %) 12 dB or worse.


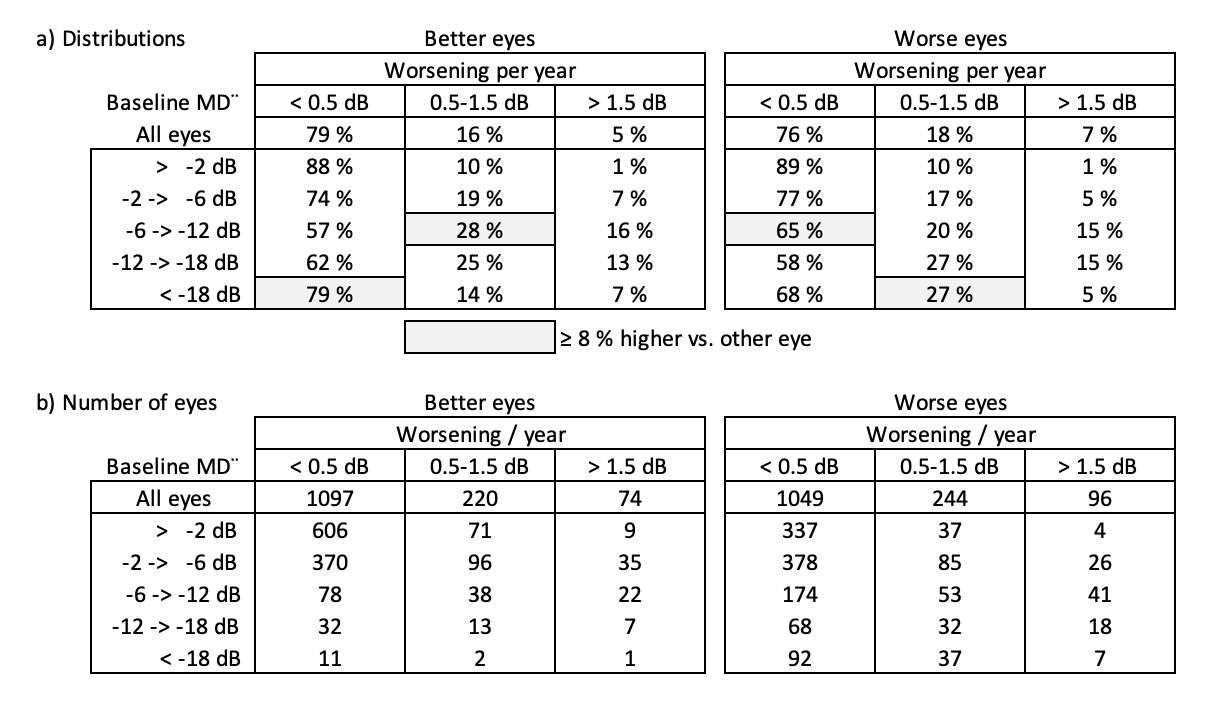

Supplement: Supplementary file 4 — Data S4: [file AOS-103-539-s004.docx]
